# Heterozygous p.S811F RET gene mutation associated with renal agenesis, oligomeganephronia and total colonic aganglionosis: a case report

**DOI:** 10.1186/s12882-016-0354-z

**Published:** 2016-10-07

**Authors:** Keisuke Sugimoto, Tomoki Miyazawa, Hitomi Nishi, Kohei Miyazaki, Takuji Enya, Mitsuru Okada, Tsukasa Takemura

**Affiliations:** Department of Pediatrics, Kindai University School of Medicine, 377-2 Ohno-higashi, Osaka-Sayama, 589-8511 Japan

**Keywords:** Intestine, Glial cell line-derived neurotrophic factor, RET, Kidney dysplasia, Nephron

## Abstract

**Background:**

Several shared common gene networks participate in development of interstinal ganglia and also nephron formation; the glial cell line-derived neurotrophic factor/Ret/glial cell line-derived neurotrophic factor receptor gene network is particularly important.

**Case presentation:**

We encountered a patient with total colonic aganglionosis as well as right renal agenesis and oligomeganephronia. Gene analysis in this patient disclosed a heterozygous p.S811F mutation was in Ret gene exon 14, resulting in a substitution of phenylalanine for serine. The large side chain of phenylalanine obstructed the opening of the hydrophobic pocket of the Ret molecule causing interference with its interaction with adenosine triphosphate and consequent marked reduction in its enzyme activity. This could account for our patient's severe intestinal disease and renal dysplasia. We know of no previous reports of concomitant Hirschsprung’s disease and oligomeganephronia.

**Conclusions:**

The patient's overall illness could be considered a novel Ret gene mutation syndrome.

## Background

Oligomeganephronia (OMN) is a type of hypoplastic kidney that most often represents congenital non-familial renal dysplasia [1]. Histopathologically, the number of nephrons per unit area is reduced, while those nephrons present show both glomerular and tubular enlargement. Unfavorable perinatal conditions such as prematurity, low birth weight, advanced maternal age, and pregnancy-induced hypertension have been reported to accompany development of OMN [1]. However, OMN can occur in the absence of such perinatal insults, arising instead from mutations affecting genes including PAX2 involved in kidney development [2, 3]. PAX2, encodes paired box gene 2, is a transcription factors expressed in nephric duct and metanephric mesenchyme and regulate the glial cell line-derived neurotrophic factor (GDNF) and/or Ret expression in the developing kidney [2]. In early phase of kidney development, the receptor of GDNF, Ret gene, and glial cell line-derived neurotrophic factor receptor (GFRα1) all are expressed throughout the region of the Wolffian duct where they take part in nephron formation under the control of GDNF [4].

In Hirschsprung’s disease (HSCR), the nerve network controlling intestinal movement is congenitally absent in the rectum and lower colon, precluding normal peristalsis and causing intestinal enlargement [5]. In addition to intestinal lesions, concomitant congenital anomalies of other organs can occur in association with HSCR, solitary kidney and renal dysplasia are among these additional disorders [6]. Association of HSCR with abnormality of the GDNF/GFRα1/Ret gene network also has been reported [7].

We encountered a patient with total-colonic aganglionosis who also had right renal agenesis and OMN.

## Case presentation

**Fig. 1:**
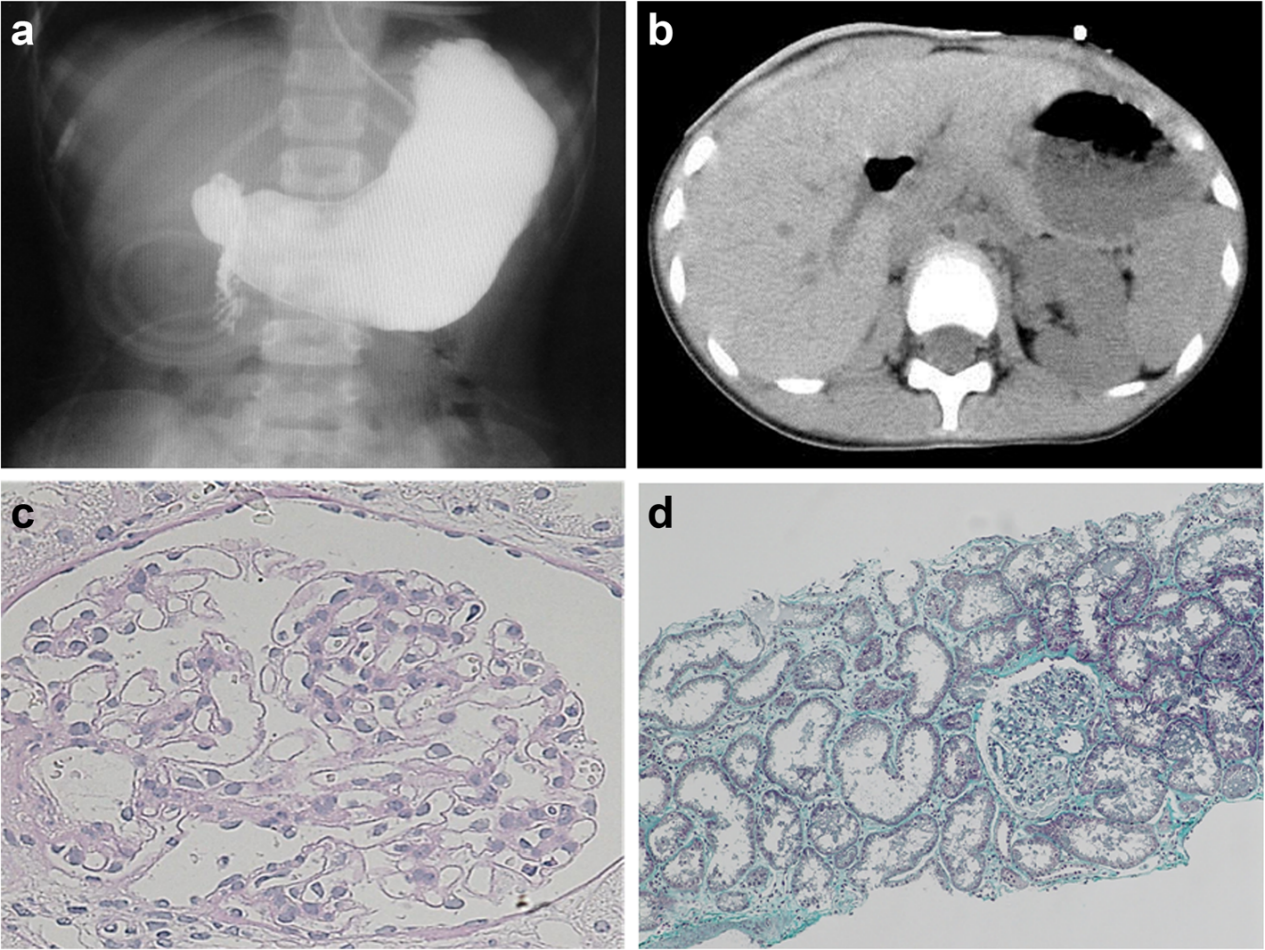
Findings in the large intestine and kidney in the present patient. Total-colon HSCR (**a**) and right renal agenesis (**b**) were present. On histologic examination of the kidney, very few glomeruli (0.96/μm2) were present, and glomeruli and renal tubules were enlarged (**c** Periodic acid-Schiff stain, x400 and **d** Masson trichrome stain, x100)

A girl, currently 11 years old, was born at 40 weeks and 3 days of gestation. Birth weight was 3148 g (+0.4 SD, relative to the mean). No known adverse perinatal condition was present. She was hospitalized for abdominal distention and bile-stained vomiting after birth. Emergency operation was done due to intestinal perforation. Diagnosed with severe total-colonic aganglionosis, she underwent total colectomy except partial jejunum and performed jejunostomy (Fig. 1a), resulting in short bowel syndrome needed permanent ostomy and treated continuously with complete intravenous nutrition. Right renal agenesis also was detected by abdominal ultrasonography after birth (Fig. 1b). Albuminuria and macroscopic hematuria appeared at about 10 years of age; urinary findings repeatedly worsened on association with upper respiratory infections. She therefore was admitted for a renal biopsy. Family history and past medical history were unrevealing.

On physical examination on admission, height was 143.8 cm (-0.2 SD); body weight, 33.8 kg (-0.6 SD); and blood pressure, 111/72 mmHg. No abnormality was noted concerning psychomotor development, nor did she show any neurologic or neuromascular abnormality. On urinalysis, urine specific gravity was 1.010, and pH was 7.5. By dipstick testing, urinary protein was 2+, and microscopic hematuria was present (3+). Microscopically, urinary red blood cell count was 50 to 99/high-power field (HPF); and white blood cell count, 1 to 4/HPF. Urinary protein level was 1.09 g/day; β2-microglobulin was 656 μg/day. Blood urea nitrogen was 29 mg/dL; serum creatinine, 1.09 mg/dL; creatinine clearance, 36.8 mL/min/1.73 m^2^; and blood cystatin C, 1.45 mg/L (normal range, 0.53 to 0.95), showing renal dysfunction. No anemia or electrolyte abnormality was detected.

Histologic findings in the renal biopsy specimen are shown in Fig. 1c and d. Four glomeruli were present. Glomeruli and renal tubules were enlarged; maximum glomerular diameter was 189.17 μm (normal range, 100 to 130), mean glomerular area was 23019 μm^2^ (normal range, 3000 to 5000) (Fig. 1c), and number of glomeruli per unit area was 0.96/μm^2^ (normal range, >5) (Fig. 1d). All of these features are characteristic of OMN. No deposition of immunoglobulin or complement was evident in glomeruli.

### Genetic analysis

**Fig. 2:**
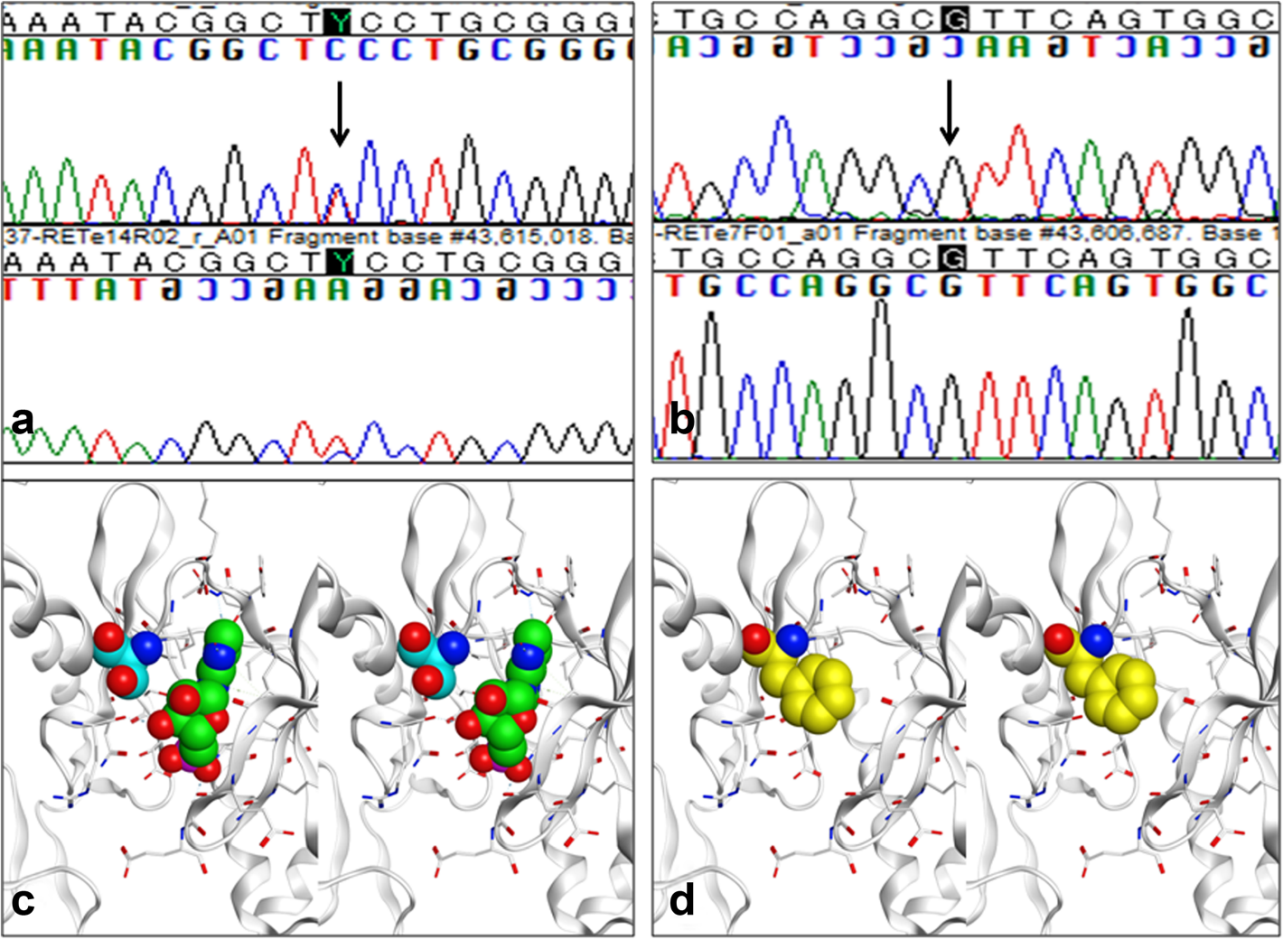
Ret gene analysis (**a** and **b**) and molecular models of the ATP binding site in the RET tyrosine kinase domain as well as a S811F mutant (**c** and **d**). In exon 14, heterozygous substitution mutation from TCC to TTC, changing serine to phenylalanine, was detected (p.S811F) (**a**, indicated by arrow), while the nucleotide at 1476 position was G (**b**, indicated by arrow). A molecular model of the ATP-binding site according to X-ray crystallography of the RET tyrosine kinase domain-AMP complex (Protein Data Bank; 2IVT) is shown in (**c**) 3-dimentionally. AMP and Ser811 respectively are represented by green and light blue spheres with some residues surrounding AMP were described by white rods in this molecular cartoon. Oxygen and nitrogen atoms are colored red and blue, respectively. An S811F mutant model was constructed based on the structure above, also is shown (**d**). Phe811 is depicted in yellow. Molecular modeling operations including display residue substitution, and structural optimization were performed using MOE 2014.09 software (Chemical Computing Group., Montreal, Canada)

Genomic DNA was extracted from peripheral blood leukocytes according to the standard protocols and performed direct sequencing by Takara Bio technology (Shiga, Japan). On analysis of GDNF/GFRα1/Ret genes, a heterozygous p.S811F mutation was detected in Ret gene exon 14 (Fig. 2a), but 1476A which is associated with small kidneys in neonates [8], was not present at position rs800, 860 of the Ret gene. Instead, the nucleotide at 1476 was G (Fig. 2b). No mutation was detected in the GDNF or PAX2 gene.

### Molecular structure analysis

The X-ray crystallographic structure of the RET tyrosine kinase domain-AMP complex [9] places Ser811 at the opening of the adenosine triphosphate (ATP) binding pocket, as shown in Fig. 2c. This hydrophobic pocket has been used as a target to accomplish chemical inhibition of RET kinase activity. In our patient's S811F mutation, the enzyme activity of RET might well be decreased, considering that ATP binding in the pocket could be obstructed by the bulky side chain of Phe811 at the opening (shown in Fig. 2d).

## Discussion

Various non-gastrointestinal disorders occur in HSCR patients, including congenital urinary tract malformations such as polycystic kidney, kidney dysplasia, vesicoureteral reflex, hydronephrosis, double ureter, and horseshoe kidney. One or more of these is evident in 20 % to 25 % of HSCR patients [6, 10].

No ganglia can be identified in the intestinal wall distal to the stomach in mice with a complete deficit of GDNF, GFRα1, or Ret [11]. This phenotype is similar to human HSCR with regard to lack of intestinal ganglia, but such extensive absence is rare in humans. In many patients, these ganglia are absent only in the lower large intestine. Importantly, development or extension of the ureteral bud does not occur in mice whose genes responsible for signal transmission via the GDNF-Ret/Gfrα1 system have been deleted [4, 12].

In Ret-deficient mice, inactivation of Ret in the late fetal period leads to absence of neural components in the lower large intestine, as also seen with GFRα1 defects, implicating reduced Ret gene expression in HSCR [13]. In mice with a lowered Ret gene expression, no abnormalities developed in the intestine or other organs in heterozygous mice whose Ret expression was decreased to 50 %. In contrast, mice whose expression was lowered to 30 %, lacked intestinal wall ganglia in distal colorectal segments; however no abnormality was noted in kidney development [13]. These findings suggest that the p.S811F mutation detected in exon 14 in our patient caused marked inhibition of Ret molecule expression or enzyme activity. We therefore analyzed the molecular structure of the Ret gene. When phenylalanine is substituted for serine, the large side chain of phenylalanine blocks the opening of the hydrophobic pocket of the Ret molecule, which may interfere with the interaction of the Ret molecule with adenosine triphosphate, markedly reducing enzyme activity that normally would prevent development of HSCR involving the entire colon, as well as right renal agenesis. Previously reported Ret gene mutations associated with total colonic aganglionosis, similar to our case, typically have been deletions or frameshifts, such as N302EfsX53, K549_G550del^D^, V636fsX1^D^, K549_G550del^D^, and V145G^D^ [14]. To our knowledge, no point mutation such as the one in our patient, has been reported to cause total colonic aganglionosis. Analysis of the specific mechanisms of pathogenesis involving the p.S811F mutation is necessary.

Uesaka et al. [13] carried out an extensive search for genes induced downstream from RET tyrosine kinase in response to GDNF stimulation using a differential display method; among the 14 genes obtained; that study identified a gene containing many zinc finger motifs at the C-terminus and at the BTB/POZ domain in the N-terminus. Antibodies prepared to investigate distribution of expression immunohistochemically confirmed that this gene is specifically expressed in the ureteric bud during development of the kidney, occurring in the RET-expressing region slightly later than the Ret expression. This suggests that expression of the gene is induced by GDNF. When those authors inhibited expression of this gene using antisense oligonucleotides in kidney organ culture, ureteric bud formation, for which the GDNF-Ret signal is essential, was greatly inhibited. This gene was named GZF1, since a GDNF-inducible gene with BTB/POZ domain and zinc finger motifs that are highly expressed in ureteric bud of the metanephric kidney; GZP1 gene is a crucial in kidney development. Our patient's p.S811F mutation may have influenced GZF1, located downstream of the GDNF/RET signaling pathway [15].

Causes of urinary abnormality and decreased renal function in our patient may involve renal pathophysiologic derangements arising from excess glomerular filtration in the left kidney as a result of right renal agenesis, as well as the paucity of nephrons imposed by OMN. When renal failure progresses, dialysis and kidney transplantation would be considered, but her underlying HSCR with short-bowel syndrome may complete these measures.

## Conclusions

Here we report a patient with total-colonic aganglionosis and concomitant right renal agenesis as well as OMN. To our knowledge, no similar case including OMN has been reported. Association of OMN with PAX2 gene mutations aberration has been reported [3], but no mutation was detected in GDNF or PAX2 in our patient. Therefore, the Ret gene mutation is likeliest cause at OMN in this individual. We consider this patient's overall disease to be a Ret gene mutation syndrome.
